# Overview of the Current Challenges in Pulmonary Coccidioidomycosis

**DOI:** 10.3390/jof10100724

**Published:** 2024-10-18

**Authors:** Mohamed A. Fayed, Timothy M. Evans, Eyad Almasri, Kathryn L. Bilello, Robert Libke, Michael W. Peterson

**Affiliations:** 1 Pulmonary Critical Care Division, University of California San Francisco, Fresno Campus, Fresno, CA 93701, USA; timothy.evans@ucsf.edu (T.M.E.); eyad.almasri@ucsf.edu (E.A.); kathryn.bilello@ucsf.edu (K.L.B.); michael.peterson3@ucsf.edu (M.W.P.); 2 Infectious Disease Division, University of California San Francisco, Fresno Campus, Fresno, CA 93701, USA; robert.libke@ucsf.edu

**Keywords:** coccidioidomycosis, valley fever, pulmonary nodules, lung mass, pyopneumothorax

## Abstract

Coccidioidomycosis is a disease caused by soil fungi of the genus *Coccidioides*, divided genetically into *Coccidioides immitis* (California isolates) and *Coccidioides posadasii* (isolates outside California). Coccidioidomycosis is transmitted through the inhalation of fungal spores, arthroconidia, which can cause disease in susceptible mammalian hosts, including humans. Coccidioidomycosis is endemic to the western part of the United States of America, including the central valley of California, Arizona, New Mexico, and parts of western Texas. Cases have been reported in other regions in different states, and endemic pockets are present in these states. The incidence of reported cases of coccidioidomycosis has notably increased since it became reportable in 1995. Clinically, the infection ranges from asymptomatic to fatal disease due to pneumonia or disseminated states. The recognition of coccidioidomycosis can be challenging, as it frequently mimics bacterial community-acquired pneumonia. The diagnosis of coccidioidomycosis is frequently dependent on serologic testing, the results of which can take several days or longer to obtain. Coccidioidomycosis continues to present challenges for clinicians, and suspected cases can be easily missed. The challenges of coccidioidomycosis disease, from presentation to diagnosis to treatment, remain a hurdle for clinicians, and further research is needed to address these challenges.

## 1. Introduction

Coccidioidomycosis, also known as San Joaquin Valley fever, is an endemic fungal infection caused by *Coccidioides* spp. These species reside in the soil of certain parts of central and southern California, the low deserts of Arizona, southeastern New Mexico, western Texas, and several other areas of the southwestern United States, Mexico, Central America, and South America [1,2]. Additionally, the endemic areas may be expanding due to climate change [3]. Residents of endemic areas are at risk of developing the infection. There are numerous presentations of the infection, ranging from asymptomatic or mild respiratory infection to severe, disseminated, fatal disease including severe acute respiratory distress syndrome (ARDS). Pulmonary infection can result in the formation of nodules that mimic lung cancer. This review focuses on the current challenges that pulmonary clinicians face in understanding the pathophysiology and immunology of coccidioidomycosis, as well as its diagnosis and clinical treatment.

## 2. Biology and Ecology

Initially, *Coccidioides* was classified as protozoan [4]. In 1905, Ophuls discovered *Coccidioides* to be a mold after identifying spherules in human tissue and arthroconidia in mycelial culture. Classification of the fungus has taken decades to mature [5]. A major change in fungal taxonomy occurred after the application of DNA fungal taxonomy and the usage of DNA sequencing [6]. *Coccidioides* is described as endemic based on the geographic distribution and classified as systemic mycosis because it causes deep-seated fungal infection. We now recognized two species: *C. immitis*, found primarily in California, and *C. posadasii*, found primarily outside California [7,8]. The two species of *Coccidioides* have asexual life cycles with distinct mold (saprobic) and spherule (parasitic) stages [9]. *Coccidioides* grow in a saprobic mycelial phase in desert environments, such as those found in North and South America. The initial growth of mycelia consists of hyphae, which septate. The septal spores degenerate and form a thin wall containing arthroconidia, which can easily be ruptured by the wind, resulting in their release [5]. The arthroconidia can either develop into mycelia in the environment again or differentiate into spherules (in mammalian hosts). Taylor, J.W., and colleagues have developed a endozoan, small-mammal reservoir hypothesis that describes the life cycle of *Coccidioides* spp. in the environment before it causes human disease. Deceased rodents that were infected with *Coccidioides* (natural death or from the disease itself) are considered to present a potential risk of spreading *Coccidioides* in the environment where the living fungi present in the deceased rodents are freed from the action of the host immune system and converted to hyphae. The hyphae grow and then produce abundant arthroconidia, which initiate a new cycle of life for the fungus [10].

### Current Challenges

- Inability to detect rodents infected with the disease to control the spread of coccidioidomycosis;
- Incomplete understanding of the environmental reservoir of *Coccidioides* in soil.

## 3. Epidemiology

In the United States, the annual incidence of coccidioidomycosis is variable, but overall is increasing [11]. According to the CDC, 10,000–20,000 cases of coccidioidomycosis is reported yearly in the United States, mostly in Arizona and California [12]. Coccidioidomycosis affects all age groups, where the highest rate was documented among adults aged between 40 and 49 [13]. Coccidioidomycosis is more common among men than women and severe or disseminated disease also occurs in men more than women [14]. Ethnic groups of Filipinos and blacks are at an increased risk of extrapulmonary complications compared to other ethnicities [15]. Exposure to disturbed soil or dust can lead to infection [16]. Populations with a significant exposure to aerosolized arthroconidia are at greater risk for infection. These groups include but are not limited to agricultural or construction workers [17,18]. Certain environmental conditions, such as wind or storms, can cause arthroconidia to be carried to different regions; therefore, people who reside near, adjacent, or travel to endemic areas are susceptible to infection [19,20].

## 4. Pathophysiology

**Figure 1:**
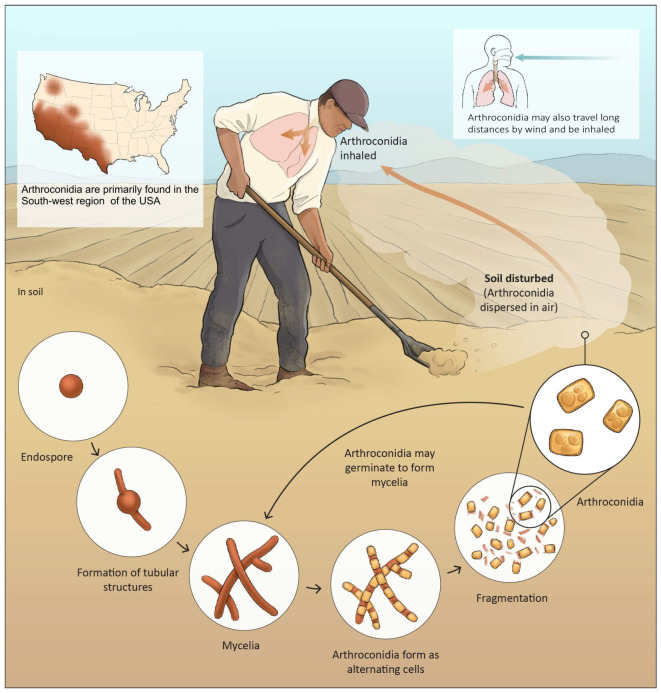
Saprobic life cycle of arthroconidia; the left-hand corner endemic map is from the CDC.

**Figure 2:**
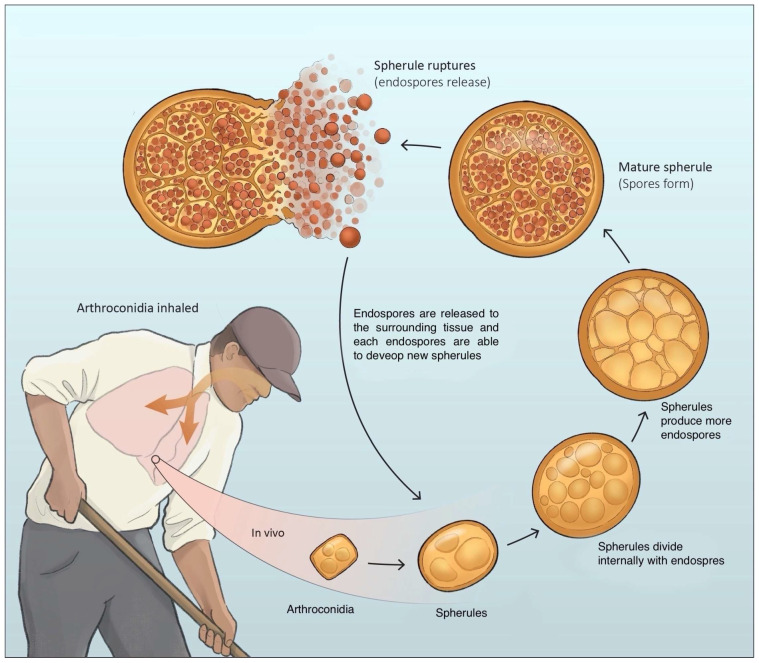
Life cycle of arthroconidia inside the lung, and typical parasitic forms of Coccidioides forming spherules and spherules/endospores.

Infection is acquired by inhalation with an incubation period of one-to-three weeks [16]. There is no minimum inoculum of exposure, and it has been suggested that a single arthroconidium can result in infection in humans [21], although more research is needed to assess whether low or high inoculum exposure can confer risk for the severity of the disease. Exposure is greatest when wind and dust conditions enhance dispersion and aerosolization of the spores and during cycles of drought and rain, which enhance fungal growth [22]. In the lungs of an infected host, a typical parasitic cycle ensues when arthroconidia transform into spherules containing multiple endospores. Upon maturation, the spherules rupture and release endospores, each of which can initiate a new spherule. Additionally, arthroconidia can produce parasitic polymorphisms and form hyphal structures that fragment into arthroconidia and differentiate into spherules, perpetuating the cycle [23]. Endospores may spread to various parts of the body, resulting in dissemination. An overview of this process is shown in Figure 1 and Figure 2.

### Current Challenges

- Difficulty in detecting arthroconidia in the air to predict outbreaks and create a preventive measure [24];
- High-risk occupations in the endemic areas have developed some preventive measures such as watering construction sites, paving roads, or planting grass, but none of these measures are supported by data [19];
- There are no robust tools to track the source of the occupational outbreaks;
- Limited guidance about high-risk postexposure prophylaxis.

## 5. Immunology of Coccidioidomycosis

**Figure 3:**
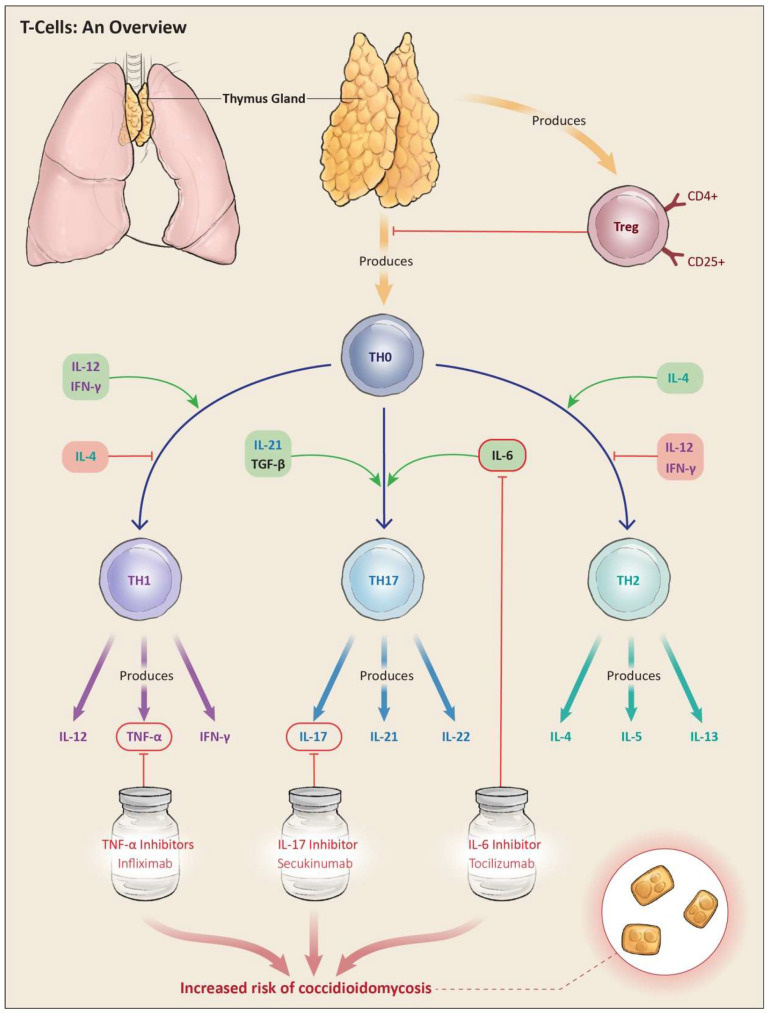
T-cell immune response to fungal infection. Th1 and Th17 are critical for the elimination and effective control of dimorphic fungi infection such as Coccidioides.

Both innate and adaptive immune responses protect humans from microbial infection. The initial protection involves the innate immune response triggered by the binding of invariant fungal elements (pathogen-associated molecular patterns [PAMPs]) to the pattern recognition receptors (PRRs) found on a variety of host cells, including dendritic cells, macrophages, neutrophils, and lymphocytes [25]. Pattern recognition receptors are a family of membrane-associated receptors that include Toll-like receptors (TLRs) and c-type lectin receptors (CLRs), among others, that help mediate the internalization and killing of microbial pathogens [25,26]. PRRs also contribute to the broader immune process through their contribution to the initiation and polarization of the adaptive immune response, controlled by lymphocytes. The innate immune response, however, is inherently limited in terms of its specificity, strength, and ability to confer immune memory [27]. In addition, the arthroconidia and spherules, which are 30–60 μm in diameter, are too large for the phagocytic mechanisms of the innate immune system [28]. The activation of the adaptive immune system occurs when T helper (Th) cells are activated through the interaction of fungal antigens with their specific T-cell surface ligands [29], and through input from the innate immune system. In particular, host dendritic cells (DRs) are central to these early events, serving as antigen-presenting cells (APCs) [29]. The development of Th1 and Th17 subtypes is particularly critical for successful protection against *Coccidioides* infection [30]. The production of tumor necrosis factor alpha (TNF-α) by Th1 cells, for example, is essential for granuloma formation in fungal infections. Under the influence of interleukin-6, interleukin-21, and tumor growth factor β (TGF-β), Th cells differentiate into Th17 cells and produce interleukin-17, a proinflammatory cytokine that is part of an important defense mechanism against fungal infection [27,28,29]. The degree to which CD8+ T-cells are involved in protection against *Coccidioides* infection is less clear, and while there is some evidence to support a protective role, more research is needed to characterize their antifungal properties. Anti-inflammatory drugs such as TNF-α inhibitors or drugs that inhibit T-cell proliferation can suppress defense mechanisms against dimorphic fungi such as *Coccidioides* [31,32,33]. See Figure 3.

### Current Challenges

- Patients who develop severe or disseminated disease without clear immunological defects are not being investigated properly in a daily clinical setting;
- There is an essential need to better understand the immune response to improve the identification of high-risk patients;
- There is unclear guidance for patients who live in endemic areas and take immunosuppressive drugs as to whether they should take chronic antifungal medication.

## 6. Clinical Manifestation of Coccidioidomycosis

Clinical manifestations of coccidioidomycosis can be divided into pulmonary and extrapulmonary. When patients do develop symptoms, they are primarily respiratory in nature; however, there are patients who present with extrapulmonary manifestations as their initial presentation [34]. Severe pulmonary and disseminated cases of coccidioidomycosis are attributed to host factors more than environmental and occupational exposures [35,36].

### 6.1. Pulmonary Coccidioidomycosis Manifestations

**Figure 4:**
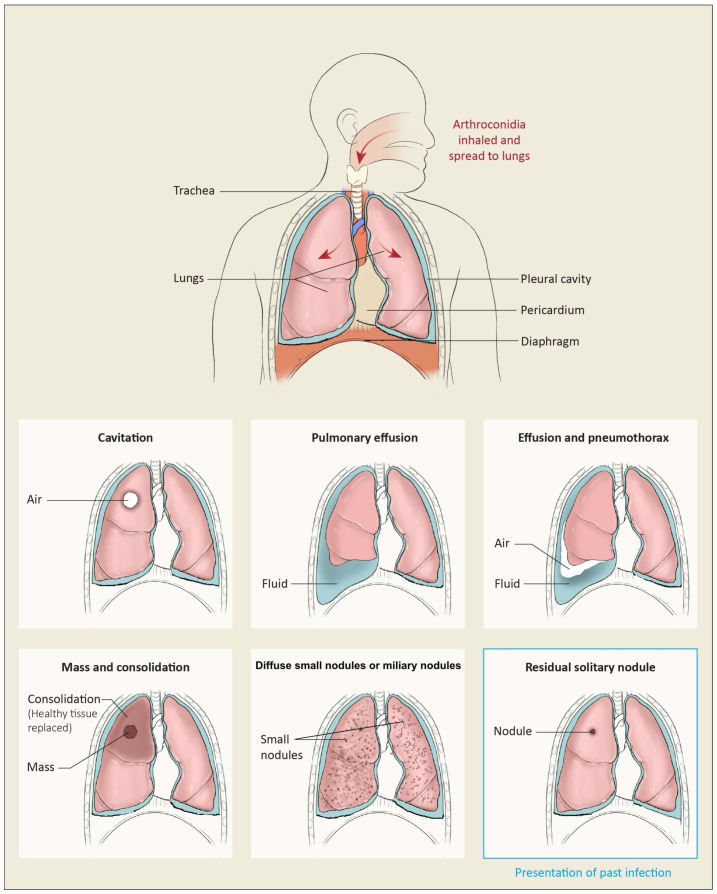
Presentation of pulmonary of coccidioidomycosis.

There are several clinical features associated with pulmonary coccidioidomycosis. Respiratory complaints are always a feature associated with generalized constitutional systemic symptoms. The systemic symptoms of headache, night sweats, weight loss, and fatigue are common [37]. Immunological symptoms can also be present, such as erythema nodosum and erythema multiforme [37]. Rheumatological symptoms such as joint pain and arthralgia without joint effusion may also be present [37]. Pulmonary presentations are outlined in Figure 4.

### 6.2. Asymptomatic or Mild Respiratory Tract Infection

It is reported that 60% of coccidioidal infections are asymptomatic or minimally symptomatic [38]. The symptoms resemble those of a common cold or lower respiratory infection: mild fever, body aches, and a mild cough [39,40]. Cases are self-limited, and patients do not seek medical attention [38]. However, this observation is derived from a report published in 1946 [38], and new studies are needed to confirm these data.

### 6.3. Acute Pneumonia Resembling Community-Acquired Pneumonia

**Figure 5:**
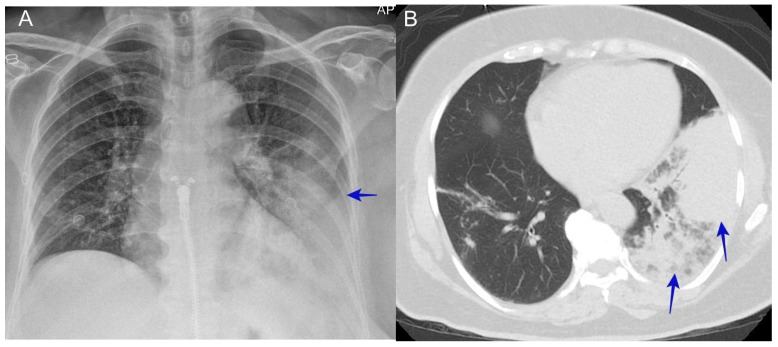
(**A**) A chest X-ray shows a left lower opacity (blue arrow). (**B**) A CT scan shows left lower lobe consolidation (blue arrows). The patient was a 65-year-old woman who presented with the chief complaint of a cough and dyspnea lasting for one week. She was diagnosed with community-acquired pneumonia and placed on IV antibiotics and sent home with oral antibiotics. She tested positive for coccidioidomycosis IgG.

The usual symptoms of acute pneumonia include a cough, fever, and the presence of infiltrates shown in chest X-rays. It is common for physicians to treat these patients for community-acquired pneumonia (CAP) with regular antibiotic therapy without testing for coccidioidomycosis and for the patient to never receive antifungal therapy [41,42]. See Figure 5.

### 6.4. Pleuritic Chest Pain and Effusion

**Figure 6:**
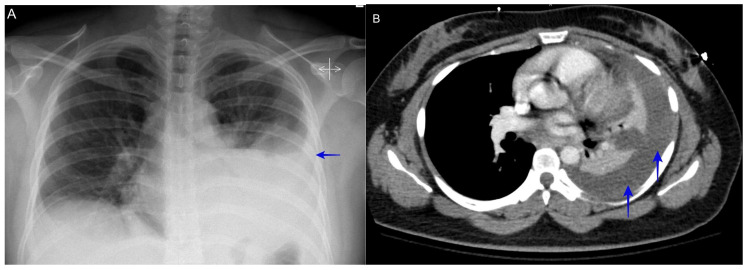
(**A**) shows a chest X-ray with left-sided pleural effusion (blue arrow). (**B**) shows a CT scan with left-sided pleural effusion (blue arrow). The patient was a 26-year-old woman who presented with progressive dyspnea and a cough associated with left-sided chest pain. She was originally diagnosed with community-acquired pneumonia until she presented to our hospital with worsening symptoms. She was diagnosed with pulmonary coccidioidomycosis via serology-positive IgM and her pleural fluid was positive for a coccidioidomycosis polymerase chain reaction (PCR).

Patients with pulmonary coccidioidomycosis often present with pleuritic chest pain or pleurisy due to the development of lung-consolidated masses. Occasionally, parapneumonic effusions can occur. The mechanism of the effusion can be a manifestation of the increased vascular permeability of the pleura due to pneumonia or a manifestation of a parenchymal lesion directly invading the pleural space, where cultures of the fluid tend to be positive for *Coccidioides* [43]. See Figure 6.

### 6.5. Lung Mass

**Figure 7:**
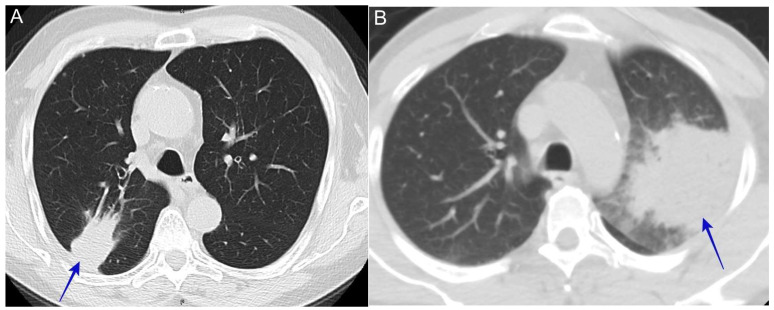
(**A**) Pulmonary coccidioidomycosis presented with a lung mass (blue arrow). The patient was a 64-year-old man who presented with a cough, shortness of breath, and night sweats and his immunodiffusion serology was positive for coccidioidal IgM and IgG. (**B**) Pulmonary coccidioidomycosis presented with a lung mass (blue arrow). The patient was a 59-year-old man with a history of diabetes who presented with 3 days of a cough, malaise, night-sweats, and left upper back pain. He underwent a bronchoscopy and the bronchioalveolar lavage culture showed *C. immitis*.

The insidious onset of systemic symptoms of weight loss, night sweats, and excessive fatigue associated with a lung mass on chest imaging can resemble malignancy. One of the common presentations at lung nodule clinics is a referral for work-up for a lung mass suspected of malignancy, possibly positive positron emission tomography (PET) scans and mediastinal adenopathy; upon the biopsy, coccidioidomycosis is discovered [44]. See Figure 7.

### 6.6. Pulmonary Cavitary Disease

**Figure 8:**
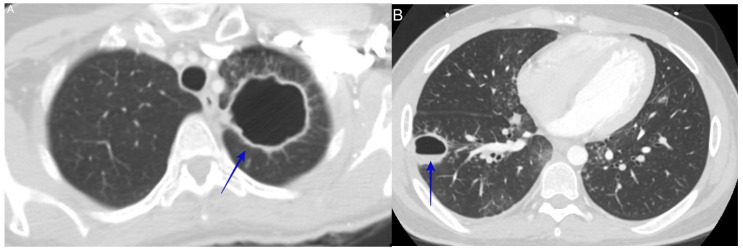
(**A**) shows a large left upper lobe cavitary lesion. The patient was a 48-year-old woman who had cavitary disease discovered during a shoulder pain work-up (blue arrow). She later tested positive for IgM and IgG coccidioidomycosis serology. (**B**) shows a right lower lobe cavitary lesion. The patient was a 22-year-old man with a history of diabetes who presented with hemoptysis (blue arrow). The fungal culture from bronchial washing showed *C. immitis*.

Patients can present with this manifestation early in the course of the primary illness or a residual cavity later in the disease course, likely because their initial symptoms were not sufficiently severe to seek care [45]. Patients typically report a chronic cough or the new onset of hemoptysis [46]. In other circumstances, this can be an incidental finding [47]. Any patients from an endemic area or who have recently traveled to an endemic area found to have cavitary disease should be promptly evaluated for coccidioidomycosis [48]. See Figure 8.

### 6.7. Severe Pneumonia with Diffuse Reticulonodular Opacities (Miliary Nodular Pattern)

**Figure 9:**
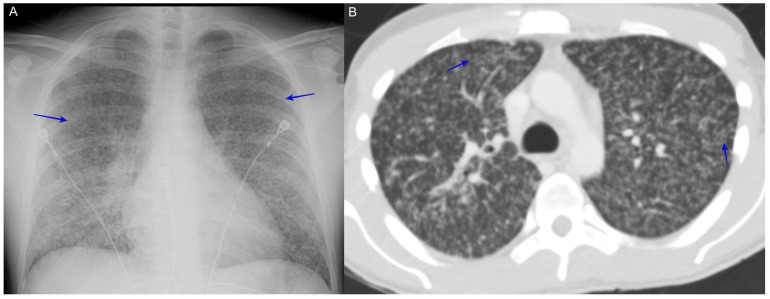
(**A**) A chest X-ray shows diffuse reticulonodular interstitial miliary nodules (blue arrows). (**B**) A CT scan shows diffuse reticulonodular interstitial miliary nodules (blue arrows). The patient was a 27-year-old man with a history of human immunodeficiency virus (HIV) non-compliant on his HIV medication who presented with a duration of cough with yellow expectoration, night sweats, chills, severe weakness, and shortness of breath upon exertion. He was diagnosed with severe coccidioidomycosis via respiratory cultures and coccidioidomycosis fungemia.

Patients present with respiratory symptoms of a cough, fever, weight loss, and night sweats. Radiologically, the appearance is similar to miliary pulmonary tuberculosis. This pattern reflects the hematogenous spread of the fungus and, in an immunocompromised host, should raise suspicion of disseminated disease [49]. See Figure 9.

### 6.8. Severe Pneumonia with Acute Respiratory Distress Syndrome (ARDS)

**Figure 10:**
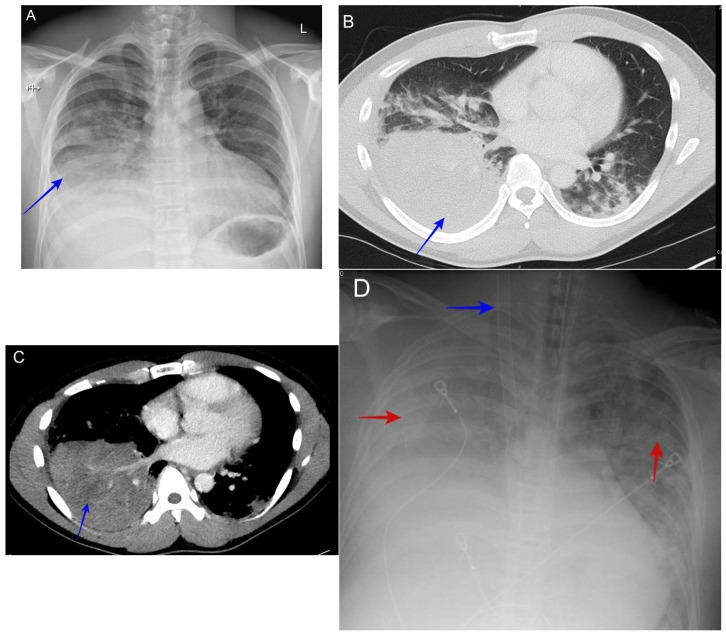
(**A**) A chest X-ray shows pulmonary coccidioidomycosis mainly involving the right lung (blue arrow). (**B**) A CT scan shows pulmonary coccidioidomycosis mainly involving the right lung with consolidation (blue arrow). (**C**) A CT scan shows pulmonary coccidioidomycosis mainly involving the right lung with consolidation and evidence of necrosis (blue arrow). (**D**) A chest X-ray shows bilateral pulmonary infiltrates (red arrows) coccidioidomycosis with progression to severe ARDS (figure on day 7). The patient was placed on extracorporeal membrane oxygenation (ECMO) therapy (blue arrow shows ECMO cannula). The patient was a 37-year-old Filipino man with no medical history who presented with a fever, cough, and right lower chest pain for 2 weeks. He was initially admitted for CAP therapy, but his cardiorespiratory status deteriorated, necessitating extracorporeal membrane oxygenation (ECMO). Respiratory cultures grew Coccidioides spp.

As with all cases of pneumonia, its severity can range from mild to severe disease with acute respiratory distress syndrome, though this is rare [50]. When it is observed, it may be seen in susceptible ethnic groups (Filipino or African American) and those with suppressed cellular immunity due to immunosuppressive medications, acquired immunodeficiency syndrome (AIDS), or pregnancy. The radiological finding is usually severe diffuse reticulonodular disease, with and without mass-like consolidation. See Figure 10.

### 6.9. Complicated Pyopneumothorax or Hydropneumothorax

The spontaneous rupture of a coccidioidomycosis pulmonary cavity resulting in pyopneumothorax or hydropneumothorax can be the initial presentation of pulmonary coccidioidomycosis [51]. The usual symptoms are excessive cough, chest pain, and dyspnea.

**Figure 11:**
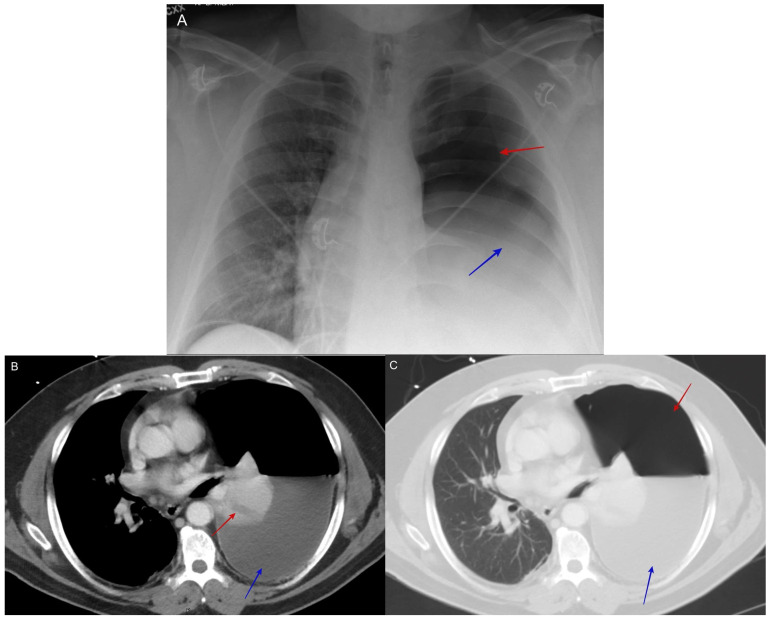
(**A**) shows the initial chest X-ray with hydropneumothorax or pyopneumothorax, (effusion blue arrow and pneumothorax red arrow). (**B**) shows the initial CT scan with hydropneumothorax or pyopneumothorax (effusion blue arrow and consolidation red arrow). (**C**) shows the initial CT scan with hydropneumothorax or pyopneumothorax (effusion blue arrow and air red arrow). The patient was a 52-year-old male construction worker who presented with a progressive cough, shortness of breath, and left-sided chest pain. He was treated as having a traumatic hydropneumothorax, then diagnosed with complicated pulmonary coccidioidomycosis based on the histopathologic exam from his decortication specimen.

Any presentation of hydropneumothorax in endemic areas without antecedent traumatic injury should prompt clinicians to suspect coccidioidomycosis. See Figure 11.

### 6.10. Lung Nodule

**Figure 12:**
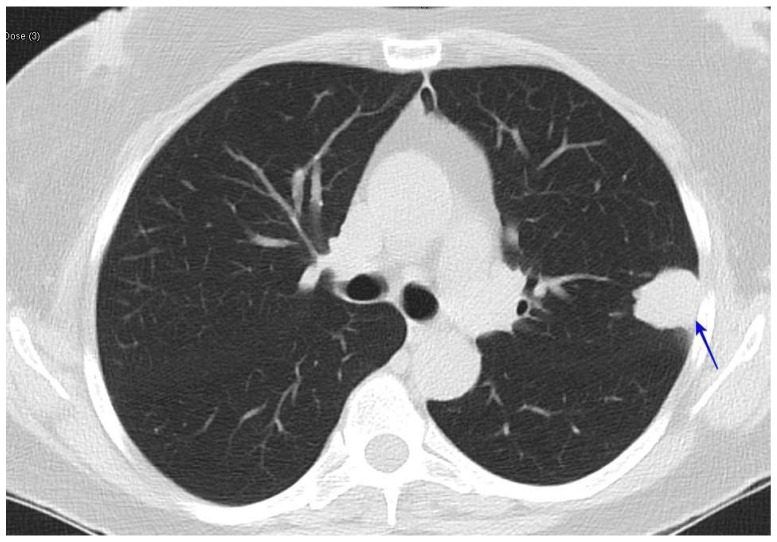
A CT scan with a left upper lobe nodule (blue arrow). The patient was a 64-year-old woman who presented with an incidental 2.5 cm left upper lobe nodule. She underwent a bronchoscopy with a biopsy, which was not diagnostic. Then, she underwent a CT guided biopsy, which showed necrotizing granuloma with fungal spherules consistent with coccidioidomycosis.

Residual nodules caused by pulmonary coccidioidomycosis pose the largest diagnostic dilemma. These nodules are discovered incidentally and can be difficult to differentiate from those due to lung cancer [52]. Some of these patients may also have risk factors for lung cancer; therefore, they may undergo extensive work-up for lung cancer [53]. In some of these cases, the patients undergo a lobectomy because the suspicion for lung cancer was very high [53]. See Figure 12.

### 6.11. Post-Coccidioidomycosis Syndrome (Fatigue)

This syndrome is defined as having persistent symptoms after the treatment of infection. The most commonly reported symptom is fatigue [54]. Symptoms of fatigue can be devastating, and on rare occasions the patient can be disabled. Other aspects of post-coccidioidomycosis are secondary to chronic infections such as chronic pulmonary coccidioidomycosis with cavitary disease.

### 6.12. Current Challenges

- Multiple different manifestations make diagnosis difficult;
- Differentiating bacterial pneumonia from coccidioidomycosis;
- Differentiated lung nodules due to *Coccidioides* vs. malignancy.

### 6.13. Extrapulmonary Manifestation

**Figure 13:**
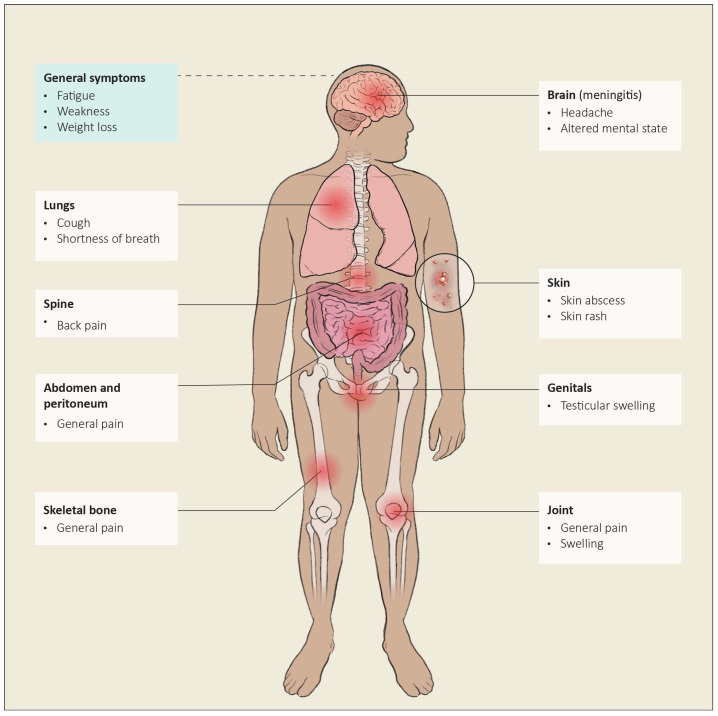
Areas of dissemination of coccidioidomycosis and/or associated symptoms.

*Coccidioides* can infect any organ. Dissemination is defined as a coccidioidal infection that presents outside the thoracic cavity. The overall incidence of dissemination is less than 1% [55,56]. The pathway of dissemination is hematogenous and lymphatic [57]. The most common locations of dissemination are skin and soft tissues, the central nervous system, the peritoneum, bones, joints, and the genital tract [58]. The most dangerous location, with an increased likelihood of a fatal outcome, is the central nervous system (CNS), where it causes meningitis [59]. The manifestation of extrapulmonary disease depends on the location of dissemination. See Figure 13.

### 6.14. Diagnostic Tools

#### 6.14.1. Serology

Serology, which is usually the initial diagnostic modality, is based on the detection of antibodies to anticoccidioidal immunoglobulin M (IgM) and immunoglobulin G (IgG). Immunocompetent patients with coccidioidomycosis show detectable serum IgM within roughly 1-to-3 weeks of symptom onset, followed by IgG production [60]. In the United States, the available serologic methods include enzyme immunoassays (EIAs), immunodiffusion (ID), complement fixation (CF), and lateral flow assays (LFAs) [60].

#### 6.14.2. Enzyme Immunoassay (EIA)

EIA is an immunological assay that incorporates the concept of an antigen binding to its specific antibody. It allows the detection of a variety of antigens or antibodies in a fluid sample and is used to detect anticoccidioidal IgM and IgG. It is a fast and simple method, and multiple EIA kits are commercially available [61]. Unfortunately, the various kits differ in their sensitivity and specificity [60]. Therefore, EIA should not be used for definitive assessment [59]. For example, if the EIA is negative but a strong clinical suspicion of coccidioidomycosis remains, then the patient should be retested using an immunodiffusion (ID) protocol [62]. Similarly, positive EIAs in areas with a low coccidioidomycosis prevalence should be confirmed by other methods, such as ID [60,63]. The use of EIA is very helpful in endemic areas because the tests are fast, and a positive test may be considered a probable infection, allowing for timely intervention [60].

#### 6.14.3. Immunodiffusion-Based (ID)

This method relies on the formation of an antigen–antibody precipitin line when an extract of *Coccidioides* antigen is tested with serum from an infected patient. In an experienced lab, this method can provide highly sensitive and specific serological testing for the detection of *Coccidioides*-specific antibodies [60]. However, the sensitivity of the ID method can vary based on multiple factors, including the duration of symptoms (e.g., early disease can have a negative serology), the patient’s immune system status (e.g., immunocompromised patients are unable to mount an antibody response), laboratory quality control, and the choice of kit; however, in clinical practice, ID is considered to be the confirmatory test after a positive EIA determination or after a negative EIA reading when a strong clinical suspicion of coccidioidomycosis remains [62]. Quantitative immunodiffusion (qID) can be used for the semiquantitative assessment of IgG. A positive serum can be serially diluted (with a 1:2 dilution factor) and plated adjacent to a well containing the antigen. After 48 h, the titer is reported as the last dilution with an observable band of precipitation [60].

#### 6.14.4. Tube Precipitin-Type (TP) Antibodies

The TP assay is one of the original methods for anticoccidioidal antibodies detection, and is reliant on the formation of an antigen–antibody precipitin when the *Coccidioides* antigen is mixed with serum from an infected patient. The TP assay is largely replaced by the current immunodiffusion [64].

#### 6.14.5. Complement Fixation (CF)

CF assay is one of the original methods for detecting coccidioidal IgG antibodies. The test relies on complement binding to antibody–antigen complexes. The test is performed when hemolysin-treated red blood cells are added to mixtures of patient serum, coccidioidal antigen, and complement [60]. Complement will lyse sensitized hemolysin-treated red blood cells (RBCs) if there are no antibody–antigen complexes (negative test). Complement will bind to antibody–antigen complexes when coccidioidal antigen–antibody complexes are present in sufficient quantities to bind all exogenous complement and, therefore, will not lyse sensitized RBCs (positive test). It is currently the standard tool for monitoring treatment response and prognosis.

CF testing provides a semiquantitative assessment of the coccidioidal IgG antibody. To evaluate the titer, positive serum samples can then be serially diluted and subjected to the same test to determine the highest concentration of patient serum that fails to lyse red blood cells. The test is performed on twofold dilutions of a single serum specimen (serial dilution with a 1:2 dilution factor). The final positive reaction is the last tube without lysed red blood cells [60].

#### 6.14.6. Lateral Flow Assay (LFA)

This test is designed to detect *Coccidioides*-specific IgM and IgG. It is a rapid test, and results can be available within 1 h. It is available commercially from IMMY (Norman, OK, USA). It possesses a poor sensitivity but high specificity [65]. Positive testing can be used to safely indicate coccidioidomycosis infection, but this should be confirmed with ID. Similarly, if it is negative and clinical suspicion is high, then ID will be the next step.

#### 6.14.7. Antigen Detection

The coccidioidal antigen detection assay is commercially available. It can be detected in body fluid such as urine and cerebrospinal fluid (CSF) as well as serum [66]. This test is usually used in immunocompromised patients when serologic testing is negative. This test is also helpful in severe diseases, particularly in coccidioidal meningitis [67].

#### 6.14.8. Cultures

Fungal culture is the gold-standard diagnostic method. The specimen for the culture is often obtained from the respiratory specimen such as sputum or bronchoalveolar lavage, but any bodily fluid can be used. Tissue cultures from a biopsy can also be sent for fungal culture. Overall, the sensitivity is poor, around 50%, and the process is lengthy [68]. *Coccidioides* is designated as a risk group level 3 organism and the culture must be carried out at biosafety level 3 (BSL-3) [69]

### 6.15. Molecular Methods

#### 6.15.1. Polymerase Chain Reaction (PCR)

The PCR is a laboratory technique that has been applied to detect *Coccidioides* in body fluid specimens. GenStat Molecular Diagnostics, LLC (St. George, UT, USA), received Food and Drug Administration (FDA) approval for the detection of *Coccidioides* spp. DNA from bronchial alveolar lavage (BAL) or bronchial wash (BW) samples [70]. Some centers perform their own PCR-based testing on bodily fluid or tissue specimens and can obtain very rapid and timely results [71]. However, the PCR is not widely available, and its sensitivity varies [72]. Our center is currently using the BD Max, a Becton Dickinson molecular instrument, which can provide results within 4 h. Our published results show 100% specificity and a variable sensitivity based on the type of clinical specimen. For example, we obtained a sensitivity of 59% for cerebrospinal fluid (CSF), 91% for BAL for acute pneumonia, 94% for sputum for acute pneumonia, and 86% for pleural fluid, whereas the sensitivity was only 44% for lung tissue biopsy from lung nodules [72]. This tool has the advantage of speed and accuracy when compared with culture methods.

#### 6.15.2. Metagenomic Next-Generation Sequencing (NGS)

NGS is a novel diagnostic tool that analyzes cell-free genetic material from a given sample without the need for predetermined sequences [73]. The sample can be used to detect hundreds of potential pathogens. Plasma microbial cell-free DNA has significant advantages because it does not require invasive sampling such as a bronchoalveolar lavage. Several reference libraries exist for these NGS tests. Some case reports showed positive results using NGS from body samples [74], as well as plasma microbial cell-free DNA [75] for testing. The turnaround time is usually at least 2 days for this method, but data are still limited, and it is considered costly.

#### 6.15.3. Pathology

**Figure 14:**
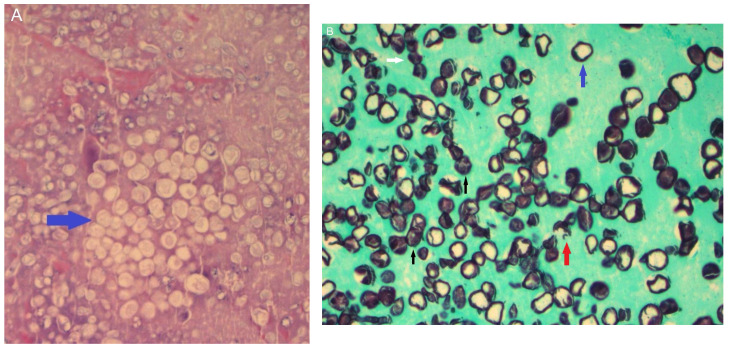
(**A**) shows spherules of Coccidioides spp. in a pleural biopsy specimen stained with hematoxylin and eosin (blue arrow). (**B**) shows a Methenamine silver stain of a pleural biopsy specimen, showing spherules (blue arrow), endospores (black arrow), the rupture of spherules and release of endospores (red arrow), and hyphal forms (white arrow). The specimens were from a 52-year-old male construction worker who presented with a progressive cough, dyspnea, and left-sided chest pain. He was diagnosed with pyopneumothorax and underwent surgical thoracotomy and decortication. A histopathologic exam of the pleural biopsy specimen showed coccidioidomycosis spherules.

Tissue samples obtained via a transcutaneous needle biopsy or endoscopic ultrasound needle biopsy can also prove invaluable in ensuring timely diagnosis. In some cases, the biopsy of lung tissue and/or lymph nodes may be the fastest way to obtain a diagnosis, although this is an invasive procedure [76]. Histopathologic exams can show if spherules have formed in the lung; sometimes, endospores are released from the ruptured spherule and develop into new spherules with necrotizing granuloma formation and eosinophilic infiltration. Other patterns that have been described in histopathologic specimens have included parasitic polymorphisms of *Coccidioides* spp., ranging from septate hyphae to hyphae composed of ovoid and spherical cells or mycelia with septate hyphae and arthroconidia. All these different histopathologic patterns are important for pathologists to recognize for accurate diagnosis (see Figure 14). A summary of the available diagnostic tools is presented in Table 1.

### 6.16. Current Challenges

- Different *Coccidioides* tests with their own sensitivity and specificity makes it challenging for physicians to order and interpret the testing;
- Lack of testing for coccidioidomycosis in outpatient care settings within a region endemic for coccidioidomycosis.

### 6.17. Making Accurate Assessments

Accurate assessment is based on two key elements. The first is suspecting the diagnosis, and the second is ordering the right testing. The use of the mnemonic C-O-C-C-I (Consider the diagnosis, Order the appropriate tests, Check for risk factors, Check for complications, and Initiate management) is also very helpful to clinicians [77].

### 6.18. Suspecting the Diagnosis

**Figure 15:**
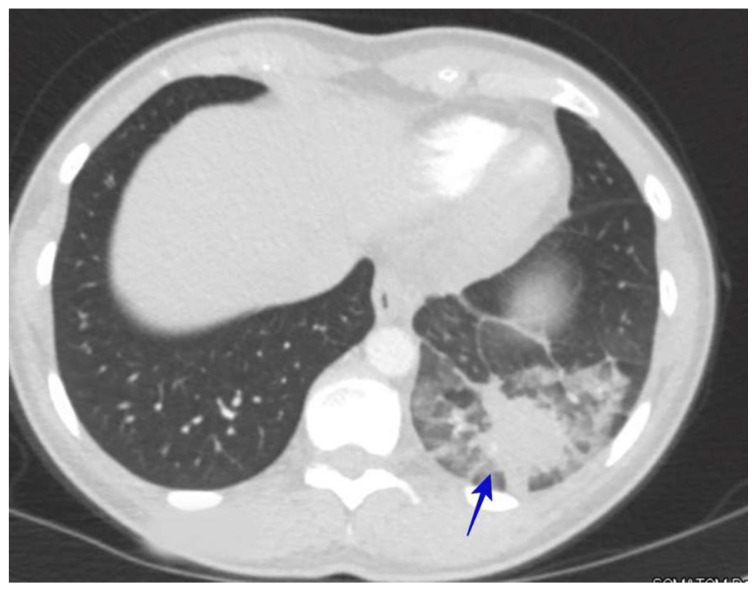
A left lower lung mass (blue arrow) that was suspected of malignancy. The mass has a subtle air bronchogram associated with ground-glass opacities and surrounding nodules. The patient was a 49-year-old patient who presented with night sweats, fatigue, and left-sided chest pain; her *Coccidioides* serologic testing for IgG was positive.

All patients residing in areas where coccidioidomycosis is endemic should be considered likely candidates for pulmonary coccidioidomycosis when they present with pneumonia. Certain key elements of the symptoms and chest images should also increase the suspicion of coccidioidomycosis among clinicians. The symptoms of coccidioidomycosis are usually more insidious than those of acute bacterial infections [55]. Excessive night sweats, weight loss, and extreme fatigue should alert clinicians to acute pulmonary coccidioidomycosis [68]. Cutaneous immunological manifestations, such as erythema nodosum and erythema multiforme, are also suggestive of coccidioidomycosis [78]. Rheumatological symptoms of multifocal arthralgias are common in acute pulmonary coccidioidomycosis. The radiological findings can be similar to those for CAP [79]; however, the presentation of lung masses with infiltration, numerous nodules, and cavitary lung disease are all suggestive of atypical pneumonia, such as that occurring with pulmonary coccidioidomycosis. Pulmonary coccidioidomycosis can present with lung masses associated with adenopathy, which can mimic lung malignancy [79,80]. Some radiographic features that suggest coccidioidomycosis infection rather than malignancy include a subtle air bronchogram associated with satellite nodules and ground-glass opacities [52]. An accurate diagnosis is critical to avoid the unnecessary use of the invasive procedures required to evaluate suspected malignancy. See Figure 15.

### 6.19. Ordering the Correct Testing

**Figure 16:**
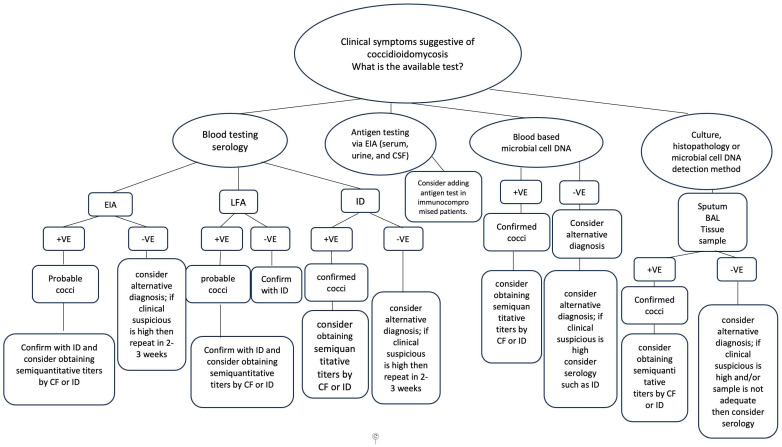
Proposed testing strategy for coccidioidomycosis. Cocci: coccidioidomycosis, EIA: enzyme immunoassay, LFA: lateral flow assay, ID: immunodiffusion, +ve: positive, −ve: negative.

Serological testing is, by far, the most common initial testing. In immunocompetent patients, coccidioidomycosis infection manifests with a detectable serum IgM within 1-to-3 weeks of symptom onset, followed by IgG production [60]. IgG production usually outlasts that of IgM and persists in chronic coccidioidomycosis. IgG can be detected in serum and other body fluids by complement fixation and immunodiffusion. The proposed testing strategy, based on the recommendations of the CDC and other experts [60,81], is outlined in Figure 16.

### 6.20. Management of Coccidioidomycosis

For pulmonary coccidioidomycosis, healthy individuals with minimal symptoms and no risk factors for complications typically do not require therapy [55]. Additionally, patients who present with incidental lung nodules and no respiratory symptoms do not require therapy. Most of the common presentations are acute CAP symptoms, and they should be managed with careful assessment based on clinical and radiological findings.

### 6.21. Identifying High-Risk Patients for Initial Therapies

Once coccidioidomycosis is confirmed, therapy should be offered to high-risk individuals who are likely to develop complications. These risk factors are related to host immune status; however, challenges arise because certain patients who develop complications and dissemination do not have typical risk factors. Ethnicity can increase the risk of disseminated disease, with a well-documented elevated risk for African American and Filipino populations [15,82]. Patients at a high risk of developing severe or disseminated coccidioidomycosis are those with a major suppression of cellular immunity, such as patients with AIDS, those with a history of solid organ or hematopoietic stem cell transplantation, and those receiving immune-suppressing agents, such as TNF-α inhibitors, high-dose glucocorticoids and other immunomodulators, or cancer chemotherapy agents associated with cytopenia [83,84,85]. Pregnant patients, when disease develops during the second or third trimester or immediately postpartum, are considered at a high risk for the development of complications [86]. Patients with diabetes, particularly those with a poor glycemic control, may increase the risk of dissemination [87]. Patients who developed multilobar infiltrate or diffuse reticulonodular pneumonia are at an increased risk of dissemination [49].

### 6.22. Medical Management of Acute Coccidioidomycosis

#### 6.22.1. Level of Care

In general, there are tools for community-acquired pneumonia that can be used as guidelines for determining the level of care (outpatient vs. inpatient). These tools include the Pneumonia Severity Index (PSI), CURB-65 (CURB-65: confusion, uremia (blood urea nitrogen > 19 mg/dL), respiratory rate > 30 breaths/min, blood pressure < 90 mm Hg, and age > 65), and SCAP (severe community-acquired pneumonia definition (major and minor)). These tools can help clinicians to determine the appropriate level of care [88,89,90].

#### 6.22.2. Outpatient Therapy (Mild or Moderate Disease)

When therapy is provided, it involves triazoles such as fluconazole (with doses ranging from 400 to 800 mg daily for 12 weeks) or itraconazole (200 mg twice daily) [55]. Patients undergoing triazole therapy require monitoring with liver function tests during therapy. The presence of comorbid conditions and/or risk factors and radiological manifestation in patients should determine the duration of therapy and the extent of monitoring. Other comorbid conditions—in particular, diabetes (and its management)—might affect the pulmonary coccidioidomycosis outcome [87]. Immunocompromised patients will need to pause use of their immunosuppressive agents if possible and diabetic patients will need to control their diabetes with the usual goal of a hemoglobin A1C of less than seven as part of their coccidioidomycosis management [87]. Chest imaging of large mass-like consolidations or symptomatic cavitary disease usually indicates prolonged therapy (from 3 months to 12 months). The duration can be shortened to 6 weeks for low-risk patients or for patients who demonstrate rapid improvement [91].

### 6.23. Inpatient Therapy (Severe Disease)

If a patient requires hospitalization because of respiratory compromise (hypoxia in room air), triazole, e.g., fluconazole, continues to be the first drug of choice. Other triazole therapies, such as posaconazole, voriconazole, or isavuconazole, can be used off-label for refractory disease [42,92]. Amphotericin agents can typically be used for patients who do not tolerate oral azole therapy or who have progressive or severe disease (lipid formulation of amphotericin B) [55]. Because of the teratogenicity of triazole therapy, amphotericin is also recommended for use during the first trimester in pregnant patients with a new diagnosis of pulmonary coccidioidomycosis [86]. The patient can be transitioned to triazole therapy for the second and third trimester. Intravenous (IV) amphotericin should not be used for coccidioidal meningitis because of its unreliable delivery to the central nervous system (CNS); however, intrathecal amphotericin can be used as an alternative agent if triazole is contraindicated or fails [55].

### 6.24. Critical Care Management (Serious or Critical Disease)

On rare occasions, patients might require admission to an intensive care unit (ICU) to manage coccidioidomycosis. The usual indication is progressive acute respiratory distress syndrome (ARDS) from coccidioidomycosis or complicated coccidioidomycosis disseminated to the CNS. ARDS is managed in the same way as other ARDS cases in terms of lung protective ventilation, as well as the usage of vasopressor support. Currently, there is a debate regarding the recommended antifungal therapy. Guidelines support the usage of amphotericin followed by triazole therapy once the patient is stable [55]. Some experts suggest the usage of combined amphotericin and triazole therapy, although there is no clear evidence of the efficacy of this approach [91]. Furthermore, there are very few case reports regarding the usage of interferon-γ for immune enhancement against fungal disease in critically ill patients with coccidioidomycosis [93]. Extracorporeal membrane oxygenation (ECMO) was used successfully in cases of refractory respiratory failure in selected cases [50]. Overall, the mortality rate of coccidioidomycosis is significantly high in the ICU, reaching more than 50% [94].

### 6.25. Current Challenges

- Clinicians lack evidence-based therapy options for patients who do not have high risk factors for complications;
- Outpatient therapy and monitoring are challenging given the paucity of specialized clinics for coccidioidomycosis;
- Data of the newer generation of triazole therapies are limited;
- There is no available therapy to boost the immune system against coccidioidomycosis in critically ill patients who develop disseminated diseases;
- Side effects and toxicity during the ICU are much more common compared to outpatient or regular inpatient therapies.

### 6.26. Surgical Management of Pulmonary Coccidioidomycosis

In most cases of pulmonary coccidioidomycosis, patients are responsive to antifungal therapy; however, surgical management can be helpful in cases where persistent symptoms or complications occur. The most common indication for surgical intervention is chronic pulmonary symptomatic cavitary disease, particularly cavitary disease, which is associated with recurrent hemoptysis [55].

### 6.27. Acute Indications for Surgical Intervention

These are rare but include pyopneumothorax, since the patient will have a bronchopleural fistula after cavity rupture [51,95]. The optimal timing for surgery is not clear, but we recommend the introduction of antifungal therapies after chest tube placement, which should be continued until the patient is stable enough for thoracotomy. Another indication for acute surgical intervention is life-threatening hemoptysis, although arteriographic embolization is usually the next step to stop the bleeding before surgical resection is considered [96].

Elective surgical intervention is used for cavitary diseases that require multiple hospitalizations for hemoptysis despite antifungal therapy. Other indications for surgical intervention include symptomatic cavitary disease with a recurrent chronic cough requiring continued antifungal therapy. Surgery can be an alternative to lifelong antifungal therapy for chronic pulmonary coccidioidomycosis [97]. Careful assessment is needed prior to surgery, including lung function tests and assessment of the localized disease areas, to determine which are suitable for resection. Diabetes is an independent risk factor for chronic pulmonary coccidioidomycosis; in particular, patients with uncontrolled diabetes with an elevated average serum glucose of more than 220 or a hemoglobin A1C of more than 9 are more likely to have symptomatic relapsing or cavitary lung disease [87].

### 6.28. Current Challenges

- Surgical intervention for pulmonary coccidioidomycosis is usually unclear to many physicians and surgeons and a lot of times the surgery is offered too early or too late;
- Lifelong antifungal therapy for chronic pulmonary coccidioidomycosis can have an alternative option, which is surgical intervention, but some of these patients have uncontrolled diabetes, which should be managed well (mainly because of the lack of available primary care) prior to surgical intervention.

### 6.29. Post-Coccidioidomycosis Sequelae Management

#### 6.29.1. Monitoring the Disease

All patients should be monitored clinically, serologically, and radiologically [55]. Symptoms monitoring, such as systemic symptoms (fatigue, weight loss, and a recurrent fever) and respiratory symptoms (cough, chest pain), are excellent tools to assess improvement. Semiquantitative testing such CF or ID can be used to monitor the disease. This can be used with or without therapy: for example, increasing titers is an indicator of progression and decreasing titers is an indication of improvement in the right clinical context [98]. CF titers greater than 1:32 is predictive of an increased likelihood of disseminated infection [99]. Repeating imaging (chest or other, depending on the disease locations) to assess improvement is usually needed but evidence-based guidance is lacking.

#### 6.29.2. Management of Incidental Lung Nodules

Lung nodule management must undergo careful assessment. Lung cancer risk factors and screening serologies for coccidioidomycosis are the main tools for clinicians during assessment. The size of the lung nodule traditionally plays a major role in decision making [100]; however, in areas where coccidioidomycosis is endemic, size might not be the best decision-making strategy, especially for low-risk groups of patients. Additionally, the usage of a positron emission tomography (PET) scan is not recommended, since coccidioidal nodules are frequently positive and cannot be used to distinguish cancerous nodules from coccidioidal nodules [101]. These coccidioidal nodules can be concerning because of their irregular shape and occasionally large size. Unfortunately, many of these nodules are diagnosed via biopsy. In many places that are not familiar with coccidioidomycosis, nodules can be treated with lobectomies as if they were early-stage lung cancer [53]. It is important to obtain a careful medical history, particularly about whether patients have been in endemic areas and regarding their occupations. Lung cancer has specific risk factors, such as a history of smoking and a family history of lung cancer; therefore, the lack of such risks in a patient should lead to a coccidioidomycosis screening serology. It is also a frequent practice in endemic areas to conduct a screening serology to rule out coccidioidomycosis before proceeding to invasive procedures. Some nodule characteristics could be very helpful to distinguish between coccidioidal nodules and malignancy [52]. One report suggests that satellite nodules and an absence of chronic lung disease had a reasonable sensitivity and specificity to predict *Coccidioides* as the cause of the nodule [52]. The duration of monitoring for a lung nodule is usually 2 years, but in the case of low-risk patients who have positive serology, it has not been clearly demonstrated that 2 years of imaging monitoring is required. If patients undergo an invasive procedure and biopsy, then confirmation of the nodule showing granuloma inflammation or the presence of spherules is diagnostic, and no further imaging is required. This strategy might be applicable to some patients to avoid unnecessary monitoring via a CT scan and ensuing exposure to radiation.

#### 6.29.3. Post-Coccidioidomycosis Fatigue Management

Persistent fatigue has been reported in the literature [54]. Antifungal therapy does not appear to influence the incidence of persistent symptoms. The current recommendations for treatment include education about disease-related fatigue and the evaluation of any other contributing factors, particularly chronic pulmonary coccidioidomycosis infection. Evaluation of sleep apnea might be needed in other cases. Strategies for managing fatigue include individualized exercise prescriptions and slow titration exercises to achieve an improvement in symptoms. Sometimes, referral to physical therapists or other exercise specialists is appropriate, based on their availability [102].

#### 6.29.4. Post-Coccidioidomycosis Psychological Impact

The diagnosis of coccidioidomycosis usually gets delayed and takes a long time [103] and can mimic lung cancer. These factors contribute to the usage of invasive procedures such as biopsies. Overall, this can affect a patient’s psychological health in endemic areas. Little has been written on this topic except on the pediatric population [104]. Counseling and valley fever support services can have positive results on these cases if it is available.

### 6.30. Current Challenges

- Commonly used lung nodule calculators for lung cancer overestimate the risk of lung cancer in areas where coccidioidomycosis is endemic [52]. Therefore, new risk stratification tools for use in these endemic areas are required;
- The natural history of lung nodules due to coccidioidomycosis is unclear and there is no clear guidance about how long it should be monitored radiologically;
- There are generally no clear guidelines for follow-up after acute infection or evidence-based approaches for managing post-coccidioidomycosis fatigue or chronic pulmonary coccidioidomycosis.

## 7. Conclusions

Progress has been slow in improving the care of patients with coccidioidomycosis disease. Although the diagnostic tools are widely available at times, it can be confusing for clinicians to choose among the numerous available tests. On the other hand, rapid diagnostic tests such as PCRs or antigen detection either are not sufficiently sensitive or have a limited availability compared to diagnostic tests for respiratory viruses. Despite this, more attention has been paid to the disease both locally and nationally. Future research must focus on prevention such as vaccination, new rapid diagnostic methods, accurate radiological assessments, and the development of new antifungal drugs.

## Figures and Tables

**Table 1:** Summary of all available testing for coccidioidomycosis.

Test	Availability	Detection
Enzyme-linked immunoassays (EIA)	Available locally in many centers in endemic areas. Available by commercial laboratories.	Anticoccidioidal antibodies IgM and IgG.
Immunodiffusion	University of California Davis lab (UC Davis). Available by commercial laboratories.	Immunodiffusion (ID) for detection of coccidioidal IgM (“precipitin”) (sometimes termed as IDTP or antibody to TP antigen). Immunodiffusion (ID) for detection of coccidioidal IgG (sometimes termed as IDCF or antibody to F antigen).
Tube precipitin (TP)-type antibodies	Largely replaced by the current immunodiffusion.	Anticoccidioidal antibodies IgM and IgG.
Lateral flow assay (LFA)	It is available commercially via IMMY.	Detects *Coccidioides*-specific IgM and IgG. It is a rapid test and results can be available within 1 h. It carries a poor sensitivity but high specificity.
Complement-fixation (CF)	Available by commercial laboratories, some California county labs, and the University of California Davis lab (UC Davis).	Measures the binding of the complement by the IgG antibody as determined by the inhibition of lysis of foreign red blood cells (the greater the dilution then the more likely the patient has extensive infection). Detects antibodies in cerebrospinal fluid.
Cultures	Available in most centers locally and commercially. It must be carried out at biosafety level 3 (BSL-3).	Cultures can be obtained from sputum, endotracheal aspirate, bronchoalveolar lavage, body fluids, and tissue.
Polymerase chain reaction (PCR)	Available locally in some medical centers and commercially via Genestate.	PCRs can be obtained from sputum, endotracheal aspirate, bronchoalveolar lavage, body fluids, and tissue.
Metagenomic next-generation sequencing (NGS)	Available locally in some medical centers and commercially: for example, Karius.	It analyzes cell-free genetic material from a given sample. It can be carried out in a body sample or serum. Sensitivity is currently unclear.
Antigen coccidioidal detection via EIA	Available commercially via ARUP and MiraVista.	Antigen in body fluids such as cerebrospinal fluid, serum, and urine.

## Data Availability

Not applicable.
